# Lateral lumbar interbody fusion (LLIF) reduces total lifetime cost compared with posterior lumbar interbody fusion (PLIF) for single-level lumbar spinal fusion surgery: a cost-utility analysis in Thailand

**DOI:** 10.1186/s13018-023-03588-w

**Published:** 2023-02-16

**Authors:** Win Boonsirikamchai, Pochamana Phisalpapra, Chayanis Kositamongkol, Ekkapoj Korwutthikulrangsri, Monchai Ruangchainikom, Werasak Sutipornpalangkul

**Affiliations:** 1 Division of Orthopaedics, Bhumibol Adulyadej Hospital, Bangkok, Thailand; 2 Department of Medicine, Faculty of Medicine Siriraj Hospital, Mahidol University, Bangkok, Thailand; 3 Department of Orthopaedic Surgery, Faculty of Medicine Siriraj Hospital, Mahidol University, Bangkok, Thailand

**Keywords:** Cost-effectiveness analysis, Cost-utility analysis, Incremental cost-effectiveness ratio (ICER), Lateral lumbar interbody fusion (LLIF), Posterior lumbar interbody fusion (PLIF), Quality-adjusted life-year (QALY)

## Abstract

**Background:**

Lumbar interbody fusion techniques treat degenerative lumbar diseases effectively. Minimally invasive lateral lumbar interbody fusion (LLIF) decreases soft tissue disruption and accelerates recovery better than standard open posterior lumbar interbody fusion (PLIF). However, the material cost of LLIF is high, especially in Thailand. The cost-effectiveness of LLIF and PLIF in developing countries is unclear. This study compared the cost-utility and clinical outcomes of LLIF and PLIF in Thailand.

**Methods:**

Data from patients with lumbar spondylosis who underwent single-level LLIF and PLIF between 2014 and 2020 were retrospectively reviewed. Preoperative and 1-year follow-up EuroQol-5D-5L and healthcare costs were collected. A cost-utility analysis with a lifetime time horizon was performed using a societal perspective. Outcomes are reported as the incremental cost-effectiveness ratio (ICER) and quality-adjusted life-year (QALY) gained. A Thai willingness-to-pay threshold of 5003 US dollars (USD) per QALY gained was used.

**Results:**

The 136 enrolled patients had a mean age of 62.26 ± 11.66 years. Fifty-nine patients underwent LLIF, while 77 underwent PLIF. The PLIF group experienced greater estimated blood loss (458.96 vs 167.03 ml; *P* < 0.001), but the LLIF group had a longer operative time (222.80 vs 194.62 min; *P* = 0.007). One year postoperatively, the groups’ Oswestry Disability Index and EuroQol-Visual Analog Scale scores were improved without statistical significance. The PLIF group had a significantly better utility score than the LLIF group (0.89 vs 0.84; *P* = 0.023). LLIF’s total lifetime cost was less than that of PLIF (30,124 and 33,003 USD). Relative to PLIF, LLIF was not cost-effective according to the Thai willingness-to-pay threshold, with an ICER of 19,359 USD per QALY gained.

**Conclusions:**

LLIF demonstrated lower total lifetime cost from a societal perspective. Regard to our data, at the 1-year follow-up, the improvement in patient quality of life was less with LLIF than with PLIF. Additionally, economic evaluation modeling based on the context of Thailand showed that LLIF was not cost-effective compared with PLIF. A strategy that facilitates the selection of patients for LLIF is required to optimize patient benefits.

## Background

Low back pain is one of the most common and expensive-to-treat causes of work-related disability in the population older than 45 years [1]. The treatment of low back pain with interlaminar lumbar instrumented fusion was validated in long-term studies, with the surgical technique showing positive outcomes [2, 3] over conservative treatment [4, 5]. Lumbar spinal fusion has become a standard surgical procedure for treating degenerative pathologies (spondylolisthesis, spinal stenosis, and degenerative disc disease). With the aging of the Thai population, lumbar spinal fusions have increased. Expenditure on the procedure has also risen with economic growth. Evaluation and improvement of the cost-effectiveness of this surgical intervention will increasingly become an area of focus for spine surgeons.

Minimally invasive surgery with lumbar interbody fusion has become an effective alternative and is increasingly used. Many new minimally invasive interbody fusion techniques have been introduced that avoid disrupting the posterior spinal musculature while providing adequate visualization and achieving surgical goals. These techniques have shortened the time to recovery and the length of hospital stay, have reduced the amount of blood lost during surgery, and have decreased the total costs. At the same time, they have provided similar health-related, quality-of-life outcomes to traditional open surgery [6–8].

Lateral lumbar interbody fusion (LLIF) has enabled surgical treatment of back and leg pain associated with lumbar spondylosis while minimizing tissue injury and accelerating recovery. Despite decreasing recovery time, this minimally invasive technique is associated with higher instrument-related costs [9]. Deluzio et al. [10] reported that the mean length of stay of patients in the LLIF group was 49% less than that for patients who underwent open posterior lumbar interbody fusion (PLIF). The researchers also reported that, compared with PLIF, LLIF provided an average cost-saving of 9.6% or 2563 US dollars (USD) per patient. In addition, Lucio et al. [9] compared the costs and outcomes of LLIF and PLIF in patients who had undergone 2-level lumbar interbody fusion. They found a shorter average length of hospital stay and a lower complication rate in the LLIF group, with an average cost-saving of 2825 USD per patient.

Previous studies on LLIF surgery demonstrated superior clinical outcomes, such as reduced time to recovery, decreased estimated blood loss, and minimized length of hospital stay relative to PLIF surgery. In addition, LLIF is considered a cost-saving or cost-effective procedure, especially in the USA [9, 10]. In Thailand, LLIF has become more prevalent than PLIF in treating lumbar spine diseases. Nevertheless, the cost-effectiveness of the procedure is still in question because of its high instrument costs [9].

This study aimed to determine the cost-utility of LLIF compared with PLIF for patients undergoing single-level lumbar fusion procedures in Thailand.

## Material and methods

This retrospective cohort study was performed by collecting data from the electronic medical records and the Siriraj Spine Registry database of Siriraj Hospital, Mahidol University, Bangkok, Thailand. The Siriraj Institutional Review Board approved the study’s protocol before the start of the research.

### Study population

Patients who underwent lumbar spinal fusion surgery with either the LLIF or open PLIF technique using one PEEK cage at a single level between 2014 and 2020 were identified. All LLIF and PLIF procedures were performed by a single surgeon (W.S.). Pedicle screw fixation for posterior supplementation was used for both the LLIF and PLIF patients. Patients were enrolled if they

- were 18 or older,
- were diagnosed with lumbar spondylosis with back and leg pain,
- underwent single-level lumbar fusion surgery with the open PLIF or LLIF technique, and
- were followed up for at least 1 year.

Operative notes, anesthesia records, discharge summaries, clinical progression notes, and cost details were collected and assessed. Patients were excluded if they had incomplete medical or surgical details, had severe postoperative complications (for example, infection, pneumonia, coronary artery disease, or stroke), or could not respond to questions effectively.

The sample size calculation was based on data from the cost-effectiveness comparison by Gandhoke et al. of single-level transforaminal lumbar interbody fusion (TLIF) and stand-alone LLIF for degenerative spondylosis [11]. Their study measured the patient-reported outcomes at the 2-year follow-up visits. The mean utilities of 45 patients who underwent single-level TLIF and 29 patients who underwent single stand-alone LLIF, measured by the EuroQoL Group’s 5-dimension (EQ-5D) questionnaire, were 0.71 ± 0.22 and 0.78 ± 0.2, respectively. After adding 10% to compensate for possible patient dropouts, the final sample size of each group in the present investigation was calculated to be 58.

### Study procedure

The patients were divided into 2 groups: an “LLIF group” and a “PLIF group.” Data relating to the preoperative, perioperative, and 1-year postoperative periods were reviewed. The estimated blood loss, operative time, and length of hospital stay were used to evaluate the perioperative outcomes. The patients’ quality of life and functional outcomes were assessed using the EuroQoL Group’s 5-dimension, 5-level (EQ-5D-5L) questionnaire and EuroQol-Visual Analog Scale (EQ-VAS) and Oswestry Disability Index (ODI) scores. The responses of EQ-5D-5L were converted to utility scores using previously published coefficient factors specific to the Thai population [12].

### Statistical analysis

The demographic and clinical characteristics of the patients were analyzed and reported descriptively. Categorical data were compared using the chi-squared test. Continuous data, all normally distributed, were compared using an independent t-test. The results are presented as the mean and standard deviation. The survival function from lifetime data was analyzed using Kaplan–Meier survival analysis. A 2-tailed probability (*P*) value of less than 0.05 was deemed statistically significant. The various data analyses were carried out with IBM SPSS Statistics for Windows, version 19.0 (IBM Corp, Armonk, NY, USA). The cost-utility analysis was performed using Microsoft Excel 2019 (Microsoft Corporation, Redmond, WA, USA).

### Economic evaluation

The cost-utility analysis compared the lifetime costs and health outcomes of the LLIF and PLIF groups. The economic evaluation followed the Consolidated Health Economic Evaluation Reporting Standards (CHEERS) statement of 2022 [13]. A decision tree and Markov model were adopted to simulate the natural history of the disease in virtual patients. We analyzed the cost-utility outcomes using a societal perspective and a lifetime time horizon following the Thai Health Technology Assessment guidelines [14]. The results are presented as incremental cost-effectiveness ratios (ICERs) in USD per quality-adjusted life-year (QALY) gained. The interpretation of the cost utility of the 2 fusion techniques was based on a willingness-to-pay (WTP) threshold of 5003 USD per QALY gained. It was derived from the Thai WTP threshold reported by the Thai Health Economic Working Group (160,000 Thai Baht (THB) per QALY gained), using the average 2022 exchange rate of 1 USD = 31.98 THB [15, 16]. An annual discount rate of 3% was used for costs and health outcomes.

### Economic model

**Fig. 1:**
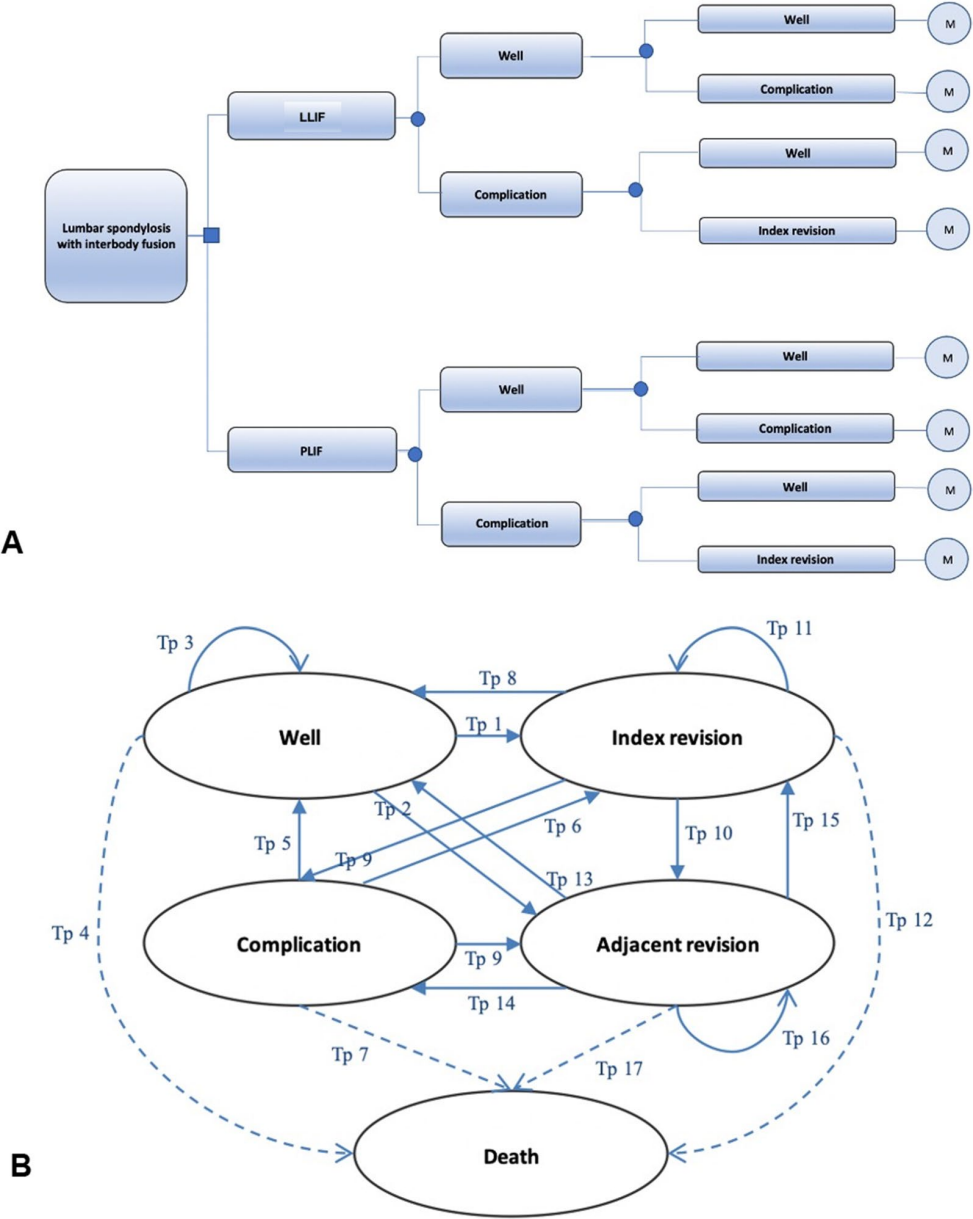
Decision tree (**A**). A decision tree was constructed to divide patients into 4 groups: “well,” “complications,” “adjacent revision,” and “index revision,” based on the health status outcomes of each treatment. Markov model (**B**). Patients could remain in the same disease state or transition to another health state

The development of our economic model commenced with the construction of a decision tree that divided patients into the LLIF and PLIF groups. As a result of our literature review and expert opinions, all health-condition outcomes were included. The decision tree subclassified the patients according to their short-term surgery outcomes (with and without complications). An index revision was performed when necessary for some complications. The tree is illustrated in Fig. 1A.

Subsequently, the patients were entered into a 5-health-state Markov model to capture the interventions’ lifetime-related costs and health outcomes. The 5 health states were “well,” “complications,” “index revision,” “adjacent revision,” and “death” (Fig. 1B). The flow of virtual patients is depicted with an arrow, with individual patients either maintaining the same disease state or transitioning to another state, per the natural course of the disease.

### Input parameters

The transitional probabilities drew data relating to disease progression and surgical techniques (LLIF and PLIF) reported by Nermani et al. [17], Nayar et al. [18], Grimm et al. [19], Kobayashi et al. [20], and Sears et al. [21]. Because our study adopted a societal perspective, the overall costs included direct medical costs (e.g., room, medication, nursing service, imaging, surgical procedure, anesthesia, implants, and physical therapy) and non-direct medical costs (such as travel and meal expenses). We assumed that a lost or impaired ability to work or engage in leisure activities due to disease morbidity and treatment would be captured as disutility in the QALY analysis [22]. Consequently, indirect costs were not included in the analysis.

The direct treatment costs, outpatient and inpatient visit rates, and utility data were obtained from the retrospective data review. Direct non-medical costs were obtained from a standard cost list that followed the Thai health technology assessment guidelines [23]. All costs were converted to 2022 USD using the consumer price index (CPI) [15, 24]. Utility data were prospectively collected at the preoperative, perioperative, and 1-year postoperative time points. Details of the input parameters used in the economic model are listed in Table 1.

**Table 1:** Input parameters used in the health economic model

Parameters	Distribution	Base case	Range	References
*Annual transition probabilities*				
Well state				
To index revision—LLIF	Beta	0.0169	0.015–0.019	[17]
To adjacent revision—LLIF	Beta	0.0088	0.008–0.010	[18]
To index revision—PLIF	Beta	0.0203	0.018–0.022	[20]
To adjacent revision—PLIF	Beta	0.0237	0.021–0.026	[21]
Complication				
To index revision—LLIF	Beta	0.0276	0.025–0.030	[19]
To index revision—PLIF	Beta	0.0203	0.018–0.022	[20]
Index revision				
To well—LLIF	Beta	0.0979	0.010–0.088	[17]
To adjacent revision—LLIF	Beta	0.0088	0.008–0.010	[18]
To well—PLIF	Beta	0.0203	0.018–0.022	[20]
To adjacent revision—PLIF	Beta	0.0237	0.021–0.026	[21]
Adjacent revision				
To well—LLIF	Beta	0.0088	0.008–0.010	[18]
To index revision—LLIF	Beta	0.0088	0.008–0.010	[18]
To well—PLIF	Beta	0.0237	0.021–0.026	[21]
To index revision—PLIF	Beta	0.0203	0.018–0.022	[20]
*Utilities*				
LLIF				
Well	Beta	0.840	0.756–0.924	Primary data
Complications	Beta	0.770	0.693–0.847	Primary data
Index revision	Beta	0.671	0.604–0.738	Primary data
Adjacent revision	Beta	0.703	0.633–0.773	Primary data
PLIF				
Well	Beta	0.890	0.890–0.979	Primary data
Complications	Beta	0.700	0.630–0.770	Primary data
Index revision	Beta	0.646	0.581–0.711	Primary data
Adjacent revision	Beta	0.679	0.680–0.747	Primary data
*Costs of treatment*				
Total cost of well—LLIF	Gamma	159	158–161	Primary data
Total cost of complication—LLIF	Gamma	244	241–246	Primary data
Total cost of Index revision—LLIF	Gamma	4041	4001–4081	Primary data
Total cost of adjacent revision—LLIF	Gamma	10 353	10,249–10,456	Primary data
Total cost of well—PLIF	Gamma	159	158–161	Primary data
Total cost of complications—PLIF	Gamma	244	241–246	Primary data
Total cost of index revision—PLIF	Gamma	4041	4001–4081	Primary data
Total cost of adjacent revision—PLIF	Gamma	5464	5410–5519	Primary data
PEEK LLIF	Gamma	1563	1548–1579	Primary data
PEEK PLIF	Gamma	563	557–568	Primary data
BMP2	Gamma	3158	3127–3190	Primary data
Posterior spinal fusion (USD)	Gamma	850	841–858	Primary data
*Direct non-medication costs*				
Food IPD (USD/visit)	Gamma	20	20–20	Primary data
Food OPD (USD/visit)	Gamma	29	28–29	[23]
Transportation (USD/visit)	Gamma	2	2–2	[23]

BMP2, bone morphologic protein 2; DM, diabetes mellitus; LLIF, lateral lumbar interbody fusion; PEEK, polyether ether ketone; PLIF, posterior lumbar interbody fusion

### Cost-utility analysis

**Fig. 2:**
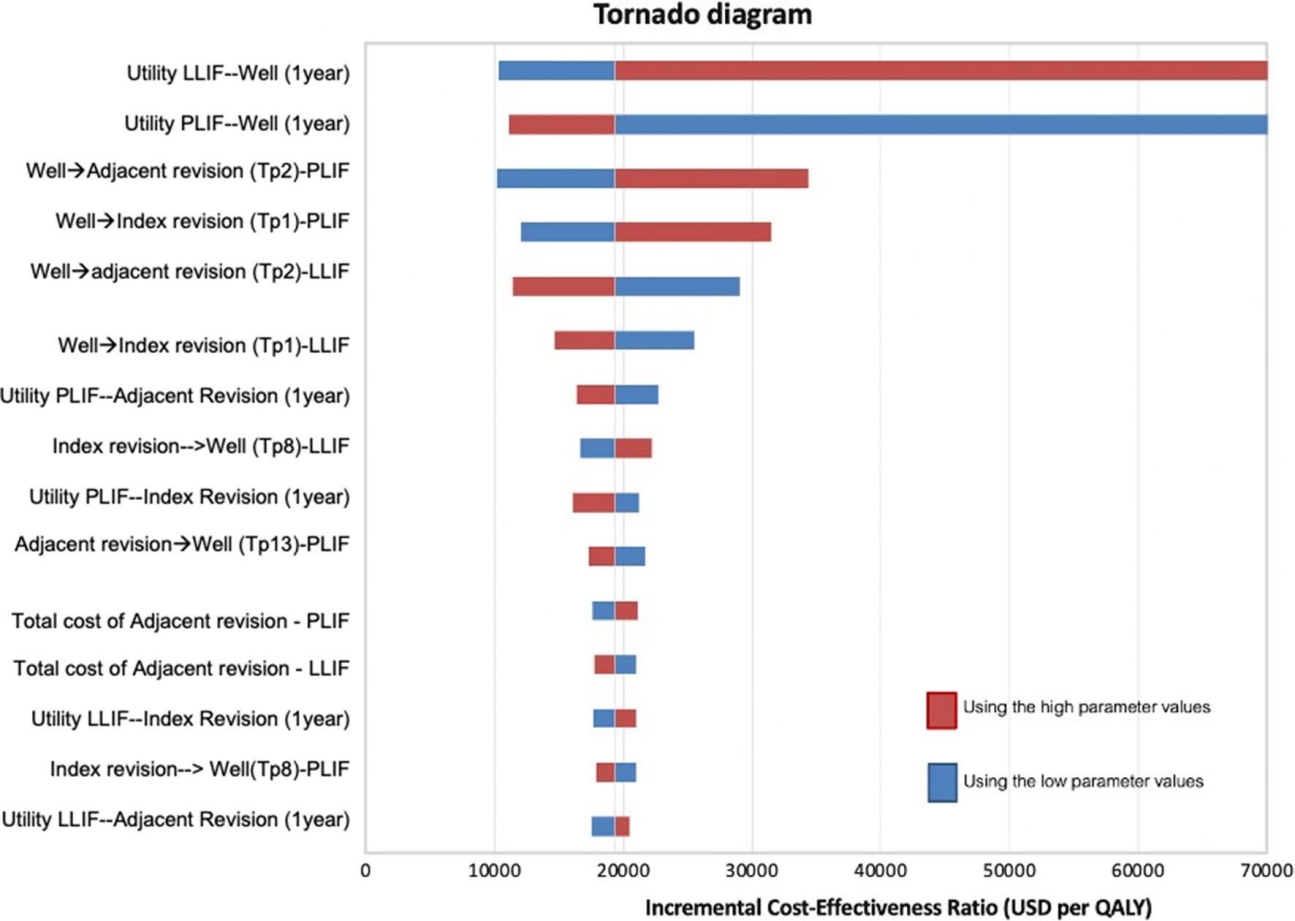
Tornado diagram. It illustrates the results of a 1-way sensitivity analysis

**Fig. 3:**
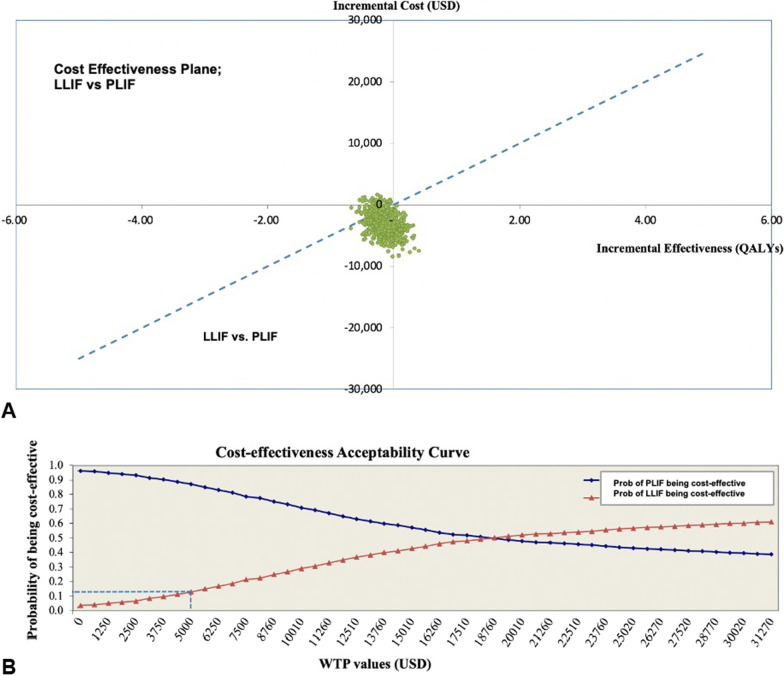
Multivariate probabilistic sensitivity analysis. The results are based on 1000 Monte Carlo simulations and are shown as a cost-effectiveness plane (**A**) and a cost-effectiveness acceptability curve (**B**)

The base-case analysis compared the health-related outcomes and costs of LLIF versus PLIF. One-way sensitivity analysis was performed to find the influence effects by altering values of the input parameters within 95% confidence interval (CI) ranges. The parameters varied in the sensitivity analysis were the clinical effects, transitional probabilities, costs, and utilities. In cases where a 95% CI range was unavailable, a range of mean ± 10% (for utility data) was applied. The results of the one-way sensitivity analysis are presented as a tornado diagram (Fig. 2). A probabilistic sensitivity analysis (PSA) was performed to examine the simultaneous effects of all parameter uncertainties. Transitional probabilities and utilities were assigned a beta distribution, while costs were assigned a gamma distribution [25]. One thousand Monte Carlo simulations were run to obtain values for total lifetime costs, outcomes, and ICERs. The PSA results are depicted as a cost-effectiveness plane and a cost-effectiveness acceptability curve (Fig. 3A, B).

## Results

### Clinical results

One hundred thirty-six spondylosis patients who underwent single-level lumbar fusion surgery were enrolled and analyzed. Their mean age was 62.26 ± 11.66 years, with 59 patients assigned to the LLIF group and 77 to the PLIF group. The patient demographic and clinical characteristics are presented in Table 2. No statistically significant differences were demonstrated in the sex, age, body mass index, underlying diseases, diagnoses, levels of operation, or length of hospital stays of the 2 groups (*P* > 0.05). Spinal stenosis, spondylolisthesis, and the L4 and L5 levels were the most common surgical treatment aspects of the 2 groups. The LLIF group experienced significantly less estimated blood loss than the PLIF group (167.03 ml vs 458.96 ml; *P* < 0.001). However, the LLIF group had a longer operative time (222.80 min vs 194.62 min; *P* = 0.007).

**Table 2:** Demographic data

Characteristics	LLIF (*n* = 59)	PLIF (*n* = 77)	*P* value
*Gender, n* (%)			
Male	17 (28.8%)	33 (42.9%)	0.092
Female	42 (71.2%)	44 (57.1%)	
Age (years) (mean + SD)	62.76 ± 11.60	61.75 ± 11.72	0.618
BMI (kg/m^2^) (mean ± SD)	25.09 ± 4.09	25.41 ± 3.81	0.642
*Underlying diseases, n* (%)			
Hypertension	32 (54.2%)	34 (44.2%)	
DM	8 (13.6%)	10 (13%)	
Coronary artery disease	4 (6.8%)	5 (6.5%)	
*Diagnosis, n (%)*			
Spinal stenosis	26 (44.1%)	32 (41.6%)	
Spondylolisthesis	28 (47.5%)	42 (54.5%)	
Degenerative disc disease	5 (8.5%)	3 (3.9%)	
*Level of spine, n (%)*			
L2–L3	3 (5%)	1 (1.3%)	
L3–L4	15 (25.4%)	5 (6.5%)	
L4–L5	41 (69.5%)	50 (64.9%)	
L5–S1	0 (0%)	21 (27.3%)	
Estimate blood loss (ml) (mean ± SD)	167.03 (161.9)	458.96 (304.36)	< 0.001*
Operative time (min) (mean ± SD)	222.8 (73.32)	194.62 (46.13)	0.007*
Length of hospital stay (days) (Mean ± SD)	8.8 (9.74)	8.34 (4.95)	0.721

BMI, body mass index; DM, diabetes mellitus; LLIF, lateral lumbar interbody fusion; PLIF, posterior lumbar interbody fusion

*Significance: *P* value less than 0.05. Statistical analysis by independent-sample *t*-test and chi-squared test

The ODI and the EQ-VAS data demonstrated improvements at the 1-year follow-up for groups, albeit without a statistically significant difference. In addition, the utility scores of both groups improved at the 1-year follow-up. However, the utility score of the PLIF group was significantly better than that of the LLIF group (0.89 vs 0.84; *P* = 0.023; Table 3).

**Table 3:** Utility, ODI, and EQ-VAS of LLIF and PLIF preoperatively and at the 1-year follow-up

Parameters	LLIF group (*n* = 59)	PLIF group (*n* = 77)	*t*	*P* value
*Preoperative*				
Utility	0.67 (0.24)	0.65 (0.17)	0.487	0.627
ODI	35.97 (19.83)	40.31 (14.56)	1.470	0.144
EQ-VAS	64.22 (19.00)	65.65 (14.27)	0.501	0.617
*1-year follow-up*				
Utility	0.84 (0.15)	0.89 (0.09)	2.307	0.023*
ODI	21.83 (15.53)	24.79 (10.28)	1.333	0.185
EQ-VAS	76.27 (18.44)	76.10 (13.44)	0.061	0.651

DM, diabetes mellitus; EQ-VAS, EuroQol-Visual Analog Scale; LLIF, lateral lumbar interbody fusion; ODI, Oswestry Disability Index; PLIF, posterior lumbar interbody fusion

*Significance: *P* value less than 0.05*.* Statistical analysis by independent-sample t-test

### Cost-utility analysis

#### Base-case analysis

The total lifetime costs per patient for LLIF versus PLIF were 30,124 USD versus 33,003 USD, respectively. Furthermore, the total QALYs were 13.48 for the LLIF group and 13.63 for the PLIF group. Taken together, these data indicated that LLIF was less costly but less effective than PLIF. The ICER for LLIF compared with PLIF was 19,359 USD per QALY gained (Table 4). This finding meant that LLIF was also not cost-effective according to the Thai WTP threshold.

**Table 4:** Results of the base-case analysis

Fusion material options	Cost (USD)	Effectiveness (QALYs)	Incremental cost (USD)	Incremental effectiveness (QALYs)	ICER (USD/QALY)
LLIF	30,124	13.48			19,359
PLIF	33,003	13.63	2878	0.15	

ICER, incremental cost-effectiveness ratio; LLIF, lateral lumbar interbody fusion; PLIF, posterior lumbar interbody fusion; QALYs, quality-adjusted life-year

#### One-way sensitivity analysis

The 3 most influential variables in our model were the utility of LLIF of the “well” health state 1 year after surgery, the utility of PLIF of the “well” health state 1 year after surgery, and the transitional probability from the “well” to the “adjacent revision” health state of PLIF (Fig. 2).

#### PSA

Based on the 1000 Monte Carlo simulations, the PSA results are presented in a cost-effectiveness plane (Fig. 3A). Despite the variations in base-case input parameters, most plots were in the left lower quadrant, suggesting that LLIF was less effective and less costly than PLIF. However, the LLIF might still be considered cost-effective in the Thai context, given that over half of the plots were under the WTP threshold line. The PSA results are also illustrated as cost-effectiveness acceptability curves (Fig. 3B). These curves demonstrated that the cost-effectiveness probability of LLIF was 13% compared with PLIF at the current Thai WTP threshold. The probability of LLIF being more cost-effective than PLIF switched at a WTP of at least 18,760 USD per QALY gained.

## Discussion

Our findings revealed that LLIF had a significantly decreased level of estimated intraoperative blood loss and significantly lower estimated total lifetime cost per patient than PLIF (30,124 USD vs 33,003 USD). In contrast, the length of hospital stay and the ODI and EQ-VAS of the LLIF and PLIF groups at the 1-year follow-up were comparable. PLIF showed a significantly better postoperative quality of life than LLIF. This finding was consistent with Gandhoke et al. [11], who reported that the utility result was higher for TLIF than for LLIF 2 years postoperatively, albeit nonsignificantly (0.67 vs 0.60; *P* = 0.96). However, our study had a larger number of samples than the investigation by Gandhoke et al., which might explain the apparent discrepancy in the statistical significance findings.

In addition, the ICER was 19,359 USD per QALY gained for LLIF compared with PLIF. According to the Thai WTP threshold (5003 USD per QALY gained), our results confirmed that LLIF was not cost-effective relative to PLIF. This significant finding may be applied to developing countries in Southeast Asia due to the health economic policy and cross-cultural similarities of the countries. Our study is the first to compare the postsurgery utility outcomes of LLIF and PLIF in a developing country.

Our key finding was the lower estimated total lifetime cost per patient for the LLIF group. This result corresponded with many previous studies that focused on cost-effectiveness evaluations of short-term postoperative periods (Table 5). Delozio et al. [10] and Lucio et al. [9] compared 2-level LLIF versus PLIF with a 45-day follow-up. They revealed that LLIF was cost-saving compared with PLIF at 2563 USD and 2825 USD per patient, respectively. Similarly, Hartman et al. [26], who compared single-level stand-alone LLIF and TLIF, revealed that stand-alone LLIF had a lower total cost (22,195 USD vs 29,953 USD) with a 30-day follow-up. When Gandhoke et al. used a 2-year follow-up to calculate the cost per person, LLIF demonstrated a higher cost (72,260 USD) than TLIF (65,179 USD). The different outcomes of Gandhoke’s study and ours might be because we used a lifetime time horizon (Gandhoke et al. evaluated the outcomes using a 2-year time horizon). An advantage of using a lifetime time horizon is that it reduces the effects of the short-term postoperative period on a model’s results.

**Table 5:** Literature review for previous studies that conducted a cost-utility analysis of lumbar interbody fusion surgery

Parameters	Deluzio et al. [10]	Lucio et al. [9]	Hartman et al. [26]	Gandhoke et al. [11]	The present study
Country	USA	USA	USA	USA	Thailand
Type of analysis	Cost-effectiveness analysis	Cost-effectiveness analysis	Cost-effectiveness analysis	Cost-utility analysis	Cost-utility analysis
Perspective	Provider	Provider	Provider	Societal	Societal
Time horizon	45 days	45 days	30 days	2 years	Lifetime
Patient data	210 patients (109 LLIF, 101 PLIF)	210 patients (109 LLIF, 101 PLIF)	20 patients (10 LLIF, 10 TLIF)	74 patients (29 LLIF, 45 TLIF)	136 patients (59 LLIF, 77 PLIF)
Costs	LLIF: 24,208 USD/patient	LLIF: 24,320 USD/patient	LLIF: 22,195 USD/patient	LLIF: 72,260 USD	LLIF: 30,124 USD
	PLIF: 26,770 USD/patient	PLIF: 27,055 USD/patient	TLIF: 29,951 USD/patient	TLIF: 65,179 USD	PLIF: 33,003 USD
	Cost-saving 2563 USD/patient	Cost-saving 2825.37 USD/patient	Cost-saving 7756 USD/patient		
Utility assessment tool	N/A	N/A	N/A	EQ-5D-5L (2 years)	EQ-5D-5L (1 year)
*Utility results*					
LLIF	N/A	N/A	N/A	0.60	0.84
PLIF	N/A	N/A	N/A	0.67	0.89
QALYs	N/A	N/A	N/A	N/A	LLIF: 13.48
					PLIF: 13.63
ICERs	N/A	N/A	N/A	LLIF: 35,347 USD/QALY	LLIF versus PLIF 19,359 USD/QALY
WTP per QALY	N/A	N/A	N/A	100,000 USD/QALY	5003 USD/QALY

Exchange rate: 1 USD = 31.98 THB^24^

ICERs, incremental cost-effectiveness ratios; LLIF, lateral lumbar interbody fusion; PLIF, posterior lumbar interbody fusion; QALY, quality-adjusted life-year; TLIF, transforaminal interbody fusion; WTP, willingness-to-pay

Based on the results of our cost-utility analysis, LLIF is not a cost-effective procedure for Thai patients. Our results correspond with the study of Gandhoke et al. [11], which conducted a cost-effectiveness analysis of single-level LLIF versus TLIF over a 2-year follow-up period. Using the short form-36 physical component summary, Oswestry Disability Index, the visual analog scale of back and leg pain, and EQ-5D, they discovered significant improvements in the LLIF and TLIF groups. The mean cumulative QALY gained by both groups were similar (0.67 vs 0.60; *P* = 0.96). The median total costs of LLIF and TLIF were 45,574 and 44,068 USD per patient, respectively (*P* = 0.96). The total costs of care and quality of life of the patients in the 2 treatment groups were very similar. Their investigation found a similar ICER outcome and a lower QALY gain 2 years postoperatively. The reduced power of indirect decompression in LLIF compared with open decompression can explain the lower quality of life. Moreover, their ICER revealed that LLIF cost an extra 35,347 USD to provide 1 additional QALY gain compared with TLIF. However, the currently commonly accepted cost-effectiveness threshold is approximately 100,000 USD in the USA. Therefore, LLIF in the study of Gandhoke et al. [11] was considered a cost-effective procedure. In contrast to our study, Hartman et al. [26] evaluated the cost-effectiveness of stand-alone lateral LLIF versus TLIF. Stand-alone LLIF was more cost-effective and offered cost-savings at the 30-day outcome.

## Strengths and limitations

There are several strengths in our study. First, our study reflects more realistic outcomes by using a lifetime time horizon to reduce the uncertainty of the outcomes of the short-term postoperative period. Second, all cost data were collected from reliable local sources, and the cost-utility analysis was conducted from a societal perspective. This approach results in reasonable outcomes that might benefit evidence-based national policy development. Contrastingly, the generalizability and transferability of the results should be remarked, due to its country-specific character. Third, we comprehensively reviewed the literature to identify relevant model input parameters. Consequently, our model drew on the most up-to-date and accurate data available.

Nevertheless, our study has some limitations. As it was retrospective, some clinical parameters were collected via a chart review. Consequently, our datasets were vulnerable to certain biases. A solid conclusion or policy decision should not be made based on this sole study. However, our study provided some potential benefit for the researchers and to be used as a part of evidence-based national policy development. It included valid model structure that have been developed via literature review and expert discussion processes. The other limitation was the potential bias on excluding patients with severe postoperative complications which may be related to the surgical techniques and result in differential costs.

## Conclusions

Lumbar spinal fusion by the LLIF technique is a less-lifetime cost procedure. Regard to our data, it improves a patient’s quality of life less at the 1-year follow-up than PLIF. In addition, LLIF is not considered cost-effective compared with PLIF at the current WTP threshold in Thailand. A strategy that helps select suitable patients for the technique may require to optimize patient benefits and develop a sustainable policy. The transferability of the results from this study should be remarked, due to its country-specific character.

## Data Availability

The data used and analyzed in this study are available from the corresponding authors on reasonable request.

## Abbreviations

EQ-5D: EuroQol-5D
EQ-VAS: EuroQol-Visual Analog Scale
ICERs: Incremental cost-effectiveness ratios
LLIF: Lateral lumbar interbody fusion
ODI: Oswestry Disability Index
PLIF: Posterior lumbar interbody fusion
QALY: Quality-adjusted life-year
TLIF: Transforaminal interbody fusion
WTP: Willingness to pay
